# Poly-γ-glutamate-based Materials for Multiple Infection Prophylaxis Possessing Versatile Coating Performance

**DOI:** 10.3390/ijms161024588

**Published:** 2015-10-15

**Authors:** Makoto Ashiuchi, Yuichi Hakumai, Shigeo Shibatani, Hirofumi Hakuba, Nogiho Oka, Hisato Kobayashi, Keizo Yoneda

**Affiliations:** 1 Graduate School of Integrated Arts and Sciences, Kochi University, Kochi 783-8502, Japan; E-Mail: b14m6f28@s.kochi-u.ac.jp; 2 Faculty of Agriculture, Kochi University, Nankoku, Kochi 783-8502, Japan; E-Mail: okanogiho@live.jp; 3 Research Center, Toyobo Co., Otsu, Shiga 520-0292, Japan; E-Mails: shigeo_shibatani@toyobo.jp (S.S.); hirohumi_hakuba@toyobo.jp (H.H.); Hisato_Kobayashi@toyobo.jp (H.K.); keizo_yoneda@toyobo.jp (K.Y.)

**Keywords:** poly-γ-glutamate, antibacterial, antiviral, coating activity, anionic polyamide, cationic surfactants

## Abstract

Poly-γ-glutamate (PGA) possesses a nylon-like backbone and polyacrylate-like carboxyl groups, and shows an extraordinary solubility in water. In this study, the effective synthesis and structural analysis of some water-insoluble PGA ion-complexes (PGAICs) using cationic surfactants, hexadecylpyridinium (HDP), dodecylpyridinium, benzalkonium and benzetonium, were examined. We demonstrated their spontaneous coating performance to the surfaces of different materials (*i.e.*, plastics, metals, and ceramics) as potent anti-staphylococcal and anti-Candida agents. The tests against *Staphylococcus aureus* revealed that, regardless of a variety of materials, PGAICs maintained surface antimicrobial activity, even after the water-soaking treatment, whereas those against *Candida albicans* indicated that, among PGAICs, PGA/HDP complex is most useful as an anti-fungal agent because of its coating stability. Moreover, the log reduction values against *Influenza A* and *B* viruses of PGA/HDP-coated surfaces were estimated to be 5.4 and 3.2, respectively, suggesting that it can be dramatically suppressed the infection of influenza. This is to our knowledge the first observation of PGA-based antiviral coatings.

## 1. Introduction

Poly-γ-glutamate (PGA), a hybrid-type polymer with a nylon-like backbone and polyacrylate-like side-chain structures, has reasonable biodegradability and good biocompatibility [1]. It meets the standards of the biochemical industry for safe use in pharmaceuticals, healthcare products, foods, and cosmetics. Because edible (stereo-irregular) dl-PGA from *natto*, a Japanese fermented food made from soybeans, significantly increases Ca^2+^ solubility *in vitro* and *in vivo* and enhances the absorption of Ca^2+^ by the intestine [2]. PGA shows promise as a therapeutic tool for osteoporosis, for instance [1]. Ashiuchi and his colleagues [3,4] recently succeeded in the plasticization of water-soluble (or hygroscopic) PGAs using a simple but effective chemical transformation, and is hereafter called the novel bio-based plastics PGA ion-complexes (PGAICs).

**Figure 1:**
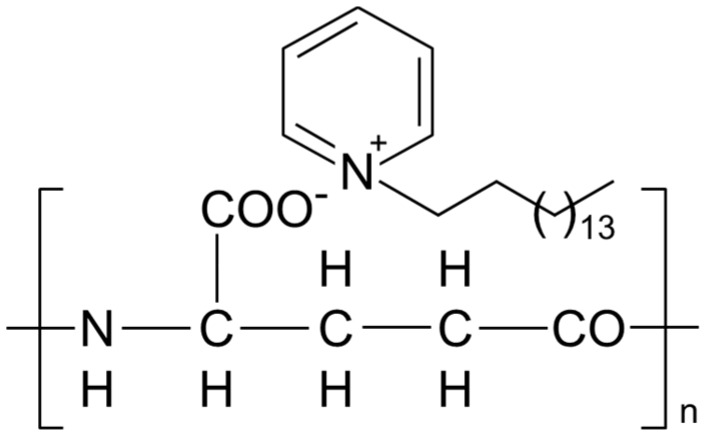
Chemical Structure of poly-γ-glutamate ion-complexes (PGAIC) consisted of PGA and hexadecylpyridinium (HDP^+^). As the molecular weight of archaeal l-PGA is on average over 800,000, its number-average degree of polymerization, *i.e.*, *n*, is estimated to be >6200.

The first PGAIC material was characterized as a stoichiometric ion-complex, containing equally the carboxyl groups of archaeal (stereo-regular) l-PGA and a compound used in toothpaste called hexadecyl-pyridinium (HDP) cation (Figure 1). This material also had the potential to serve as a functional plastic showing a broad spectrum of antimicrobial activity (against food-poisoning bacteria, a prevalent species of Candida, and filamentous fungi); this is specifically useful to the food-related engineering and pharmaceutics [3]. However, it is still unclear whether or not the extreme hydrophilicity of PGA can be suppressed using other cationic surfactant-candidates than HDP^+^, e.g., dodecylpyridinium (DDP), benz-alkonium (BZA), and benzetonium (BZT) cations, to form the water-insoluble PGAIC materials, as well as further examinations on the functionality of PGAICs for application in hygiene technology.

Here, we present the synthesis and structural analysis of new PGAIC materials using DDP^+^, BZA^+^ and BZT^+^, instead of HDP^+^, and their spontaneous coating performance to various material surfaces as anti-microbial agents. This study also reveals that PGAIC is a promising candidate for anti-influenza coatings.

## 2. Results and Discussion

### 2.1. Synthesis and Structure of New Poly-γ-glutamate Ion-Complexes (PGAIC) Materials

**Figure 2:**
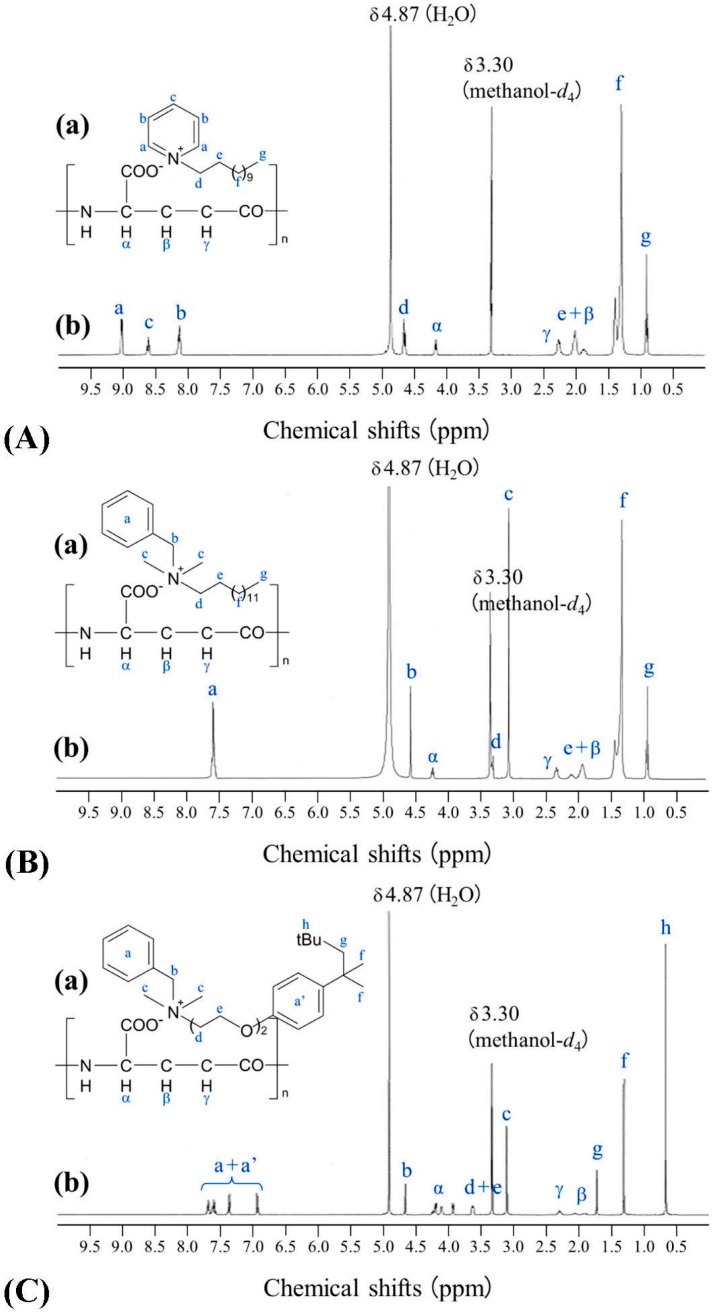
(**a**) Predicted chemical structures and (**b**) liquid ^1^H-NMR analysis of new PGAICs. (**A**) PGA/dodecylpyridinium (DDP); (**B**) PGA/benz-alkonium (BZA); and (**C**) PGA/benzetonium (BZT).

We first report the effective transformation of l-PGA using DDP^+^, BZA^+^ and BZT^+^, instead of HDP^+^; in the experiment, little or no l-PGA (<1 wt % of the initial amounts; *n* = 5) was detected in the aqueous phase of the reaction mixtures using published methods [5], indicating that PGA was almost completely transformed into a water-insoluble poly-ionic complex by interacting with the monomer molecules of HDP^+^, DDP^+^, BZA^+^, and BZT^+^. These are described as PGA/HDP (Figure 1), PGA/DDP, PGA/BZA, and PGA/BZT, respectively. Then, the structural features of PGA/DDP, PGA/BZA, and PGA/BZT were ascertained via ^1^H-nuclear magnetic resonance (NMR) analysis (Figure 2). These showed that all the PGAICs comprised equal numbers of carboxyl groups from PGA and the partner surfactants (see Supplementary Section), similarly to the first report on the molecular structure of PGA/HDP [3].

Although these PGAIC materials never swell in water, they can exhibit good solubility in alcohols and chloroforms. In the solvation properties, new PGAICs were obviously different from deprotonated PGA and covalently cross-linked PGA hydrogels [1], but resembled PGA/HDP plastics [3]. All the PGAICs were insoluble in dimethyl sulfoxide, differently from the partner surfactants and protonated PGA. Gel permeation chromatography (GPC) of PGAICs was then tried in a mobile phase of ethanol (Figure S1).

### 2.2. Versatile Coating Performance of PGAICs

**Figure 3:**
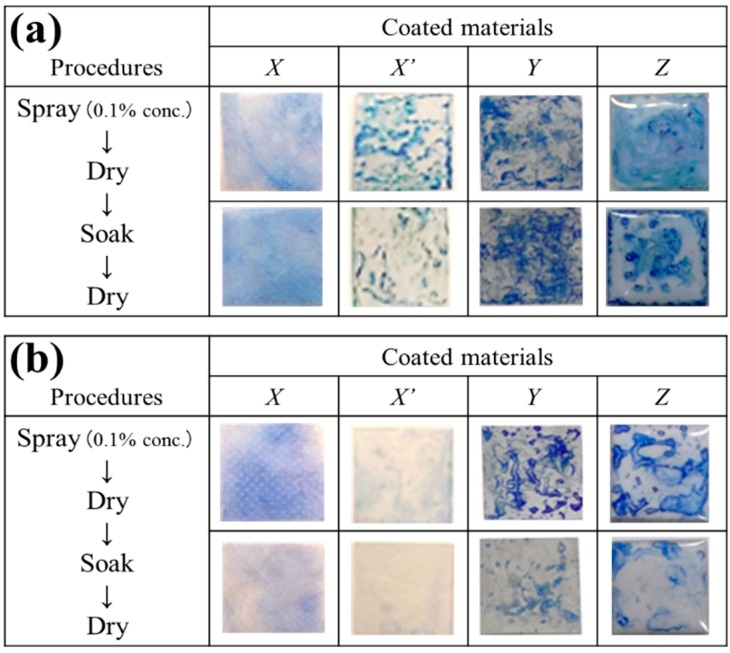
Bromophenol blue (BPB) staining of (**a**) PGA/HDP- and (**b**) *n*-Octadecyldimethyl (3-(trimethoxysilyl) propyl) ammonium (QAS)-coated surfaces. Coated materials: images *X*, a PP disk; *X’*, a PET film; *Y*, a stainless steel sheet; *Z*, a bathroom tile sheet. In (**a**,**b**), the upper and lower images were obtained using the coated materials before and after the water-soaking treatment, respectively. The data indicate that the PGAIC is superior to QAS in the coating performance (particularly on PET surfaces) and versatility.

Because PGA has potential for use as surface-contact adhesives [1], we were interested in discovering whether or not PGAIC retains the original function of PGA. Some material surfaces-coating tests were carried out using PGAICs dissolved in ethanol (0.1 wt % each), and the solvent that contains only 0.1 wt % partner surfactant was used as control. In the first experiment, the PGA/HDP-, PGA/DDP-, PGA/BZA-, PGA/BZT-, and partner surfactants-coated polypropylene (PP) disks were placed on every bed of a pathogenic bacterium (*S. aureus*; Figure S2a) and a prevalent species of Candida (*C. albicans*; Figure S2b) in an agar plate. The assessment of antimicrobial performance was based on the presence of the zone of growth inhibition (also known as halos) around the disks (Table 1). Next, the PGAIC- and partner surfactant-coated stainless steel (Figure S3) and bathroom tile (Figure S4) sheets were used for the tests, and their anti-staphylococcal (Table 1a) and anti-fungal activities (Table 1b) were assayed as well. The results from the tests against *S. aureus* revealed that, regardless of a variety of materials, PGAICs retained surface antimicrobial activity, even after the water-soaking treatment. It is well known that organosilicon-type immobilizing surfactants, e.g., *n*-octadecyldimethyl (3-(trimethoxysilyl) propyl) ammonium (QAS) cation (Figure S5a), are covalently bound to glass and cotton by the silyl group and introduce a significant anti-staphylococcal activity [6]. Nevertheless, the anti-staphylococcal activity of QAS-coated materials (*viz.*, plastics, metals, and ceramics) could not be demonstrated under the experimental conditions (Figure S5b), implying the limited performance of antimicrobial coatings concomitantly with chemical (covalent) modification of material surfaces. QAS-coated surfaces, particularly after the water-soaking treatment, were actually hard to be stained with acidic dyes including a bromophenol blue (BPB), compared with PGAIC-coated surfaces (Figure 3). QAS could be more easily released from material surfaces without additional covalent linkages. Conversely, these reveal the potentiality of PGAICs as potent, stable, widely applicable agents where neither chemical modification (e.g., non-biodegradable crosslinking) nor thermal treatment (e.g., heating) is required in the coating process. We further found the inhomogeneous distribution of PGAICs and QAS on the materials (Figure 3), implying the relativity between the behavior of spontaneous adhesion in coating agents and the surface roughness on base materials. To date, it remains to fully understand the surface-adhesion mechanism of PGAICs and to establish a feasible strategy for homogeneous coating of PGAICs, in addition to a most practical (spray) method handled in this study.

**Table 1:** Halo assays for determination of (**a**) anti-staphylococcal and (**b**) anti-Candida activities of PGAIC-coated materials *^a^*^,*b*^.

**(a)**	**Water-Soaking**	**Coating Materials**							
**Base Materials**	**Treatment**	**PGA/HDP**	**HDP^+^**	**PGA/DDP**	**DDP^+^**	**PGA/BZA**	**BZA^+^**	**PGA/BZT**	**BZT^+^**
PP disks	Before	+ (1.0)	+ (1.6)	+ (5.8)	+ (8.4)	+ (5.4)	+ (7.2)	+ (4.8)	+ (4.6)
	After	+ (1.2)	—	+ (7.6)	—	+ (7.0)	—	+ (4.6)	—
Stainless steel sheets	Before	+ (1.8)	+ (1.6)	+ (5.0)	+ (3.6)	+ (3.6)	+ (3.6)	+ (4.2)	+ (4.2)
	After	+ (1.6)	—	+ (3.0)	—	+ (2.4)	—	+ (2.6)	—
Bathroom tile sheets	Before	+ (2.0)	+ (1.6)	+ (9.4)	+ (3.6)	+ (5.2)	+ (3.6)	+ (6.2)	+ (4.2)
	After	+ (2.0)	—	+ (7.6)	—	+ (3.8)	—	+ (4.2)	—

**(b)**	**Water-Soaking**	**Coating Materials**							
**Base Materials**	**Treatment**	**PGA/HDP**	**HDP^+^**	**PGA/DDP**	**DDP^+^**	**PGA/BZA**	**BZA^+^**	**PGA/BZT**	**BZT^+^**
PP disks	Before	+ (0.1)	+ (1.0)	—	—	+ (0.2)	+ (3.6)	+ (1.2)	+ (4.8)
	After	+ (0.3)	—	—	—	+ (0.2)	—	+ (1.4)	—
Stainless steel sheets	Before	+ (0.3)	+ (<0.1)	—	—	+ (0.1)	+ (0.6)	+ (0.1)	+ (0.7)
	After	+ (0.3)	—	—	—	—	—	—	—
Bathroom tile sheets	Before	+ (0.4)	+ (<0.1)	—	—	+ (<0.1)	+ (0.4)	+ (0.9)	+ (0.7)
	After	+ (0.4)	—	—	—	—	—	+ (0.6)	—

*^a^* Symbols +, positive (on the results that halos are present around the coated materials); —, negative (on the results that halos are present around the coated materials); *^b^* The average widths (mm; *n* = 3) of halos around the materials were indicated in parentheses.

Furthermore, the results from the tests against *C. albicans* indicated that, among PGAICs, PGA/HDP (Figure 1) is most useful as an anti-fungal agent because it shows a good coating stability for plastics, metals, and ceramics (Table 1b). The zone of inhibition, however, was not found around PGA/DDP- and DDP^+^-coated materials. This is presumably due to their lower anti-Candida activities, indicated from the assay of minimal inhibition concentrations against microbial colony formation [7,8] (Table 2), while PGA/DDP may show a specific potential as an anti-Pseudomonas agent in therapeutic coating (Table 2).

**Table 2:** Minimal inhibition concentrations (MICs) of PGAICs, partner cationic surfactants, and an organosilicon-type immobilizing surfactant QAS *^a^*.

	Tested Materials								
**Microorganisms**	**PGA/HDP**	**HDP^+^**	**PGA/DDP**	**DDP^+^**	**PGA/BZA**	**BZA^+^**	**PGA/BZT**	**BZT^+^**	**QAS^+^**
*Staphylococcus aureus*	4	2	48	24	8	8	64	64	>200
*Escherichia coli*	100	64	200	128	100	100	100	100	>500
*Pseudomonas aeruginosa*	>500	>500	100	64	400	400	200	200	>500
*Candida albicans*	50	25	128	100	25	25	64	64	>500

*^a^* Concentration, ppm.

### 2.3. Anti-Influenza Activity of PGAIC

**Figure 4:**
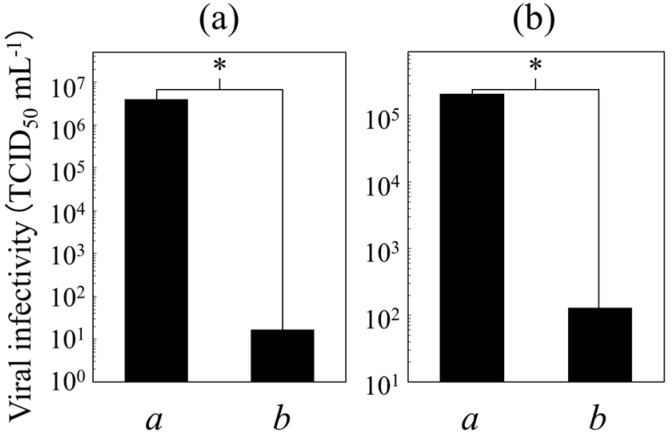
Anti-infection performance on (bars *a*) non-coated and (bars *b*) PGA/HDP-coated PET films against (**a**) *Influenza A* (H1N1, A/PR/8/34) and (**b**) *B* (B/Shanghai/361/2002) viruses. The viral infectivity was defined as a median tissue culture infectious dose (TCID_50_) [10] per mL of the sample. Asterisks (*): the significance in virus inactivation on the assessment using the log reduction values (LRV) scores.

**Figure 5:**
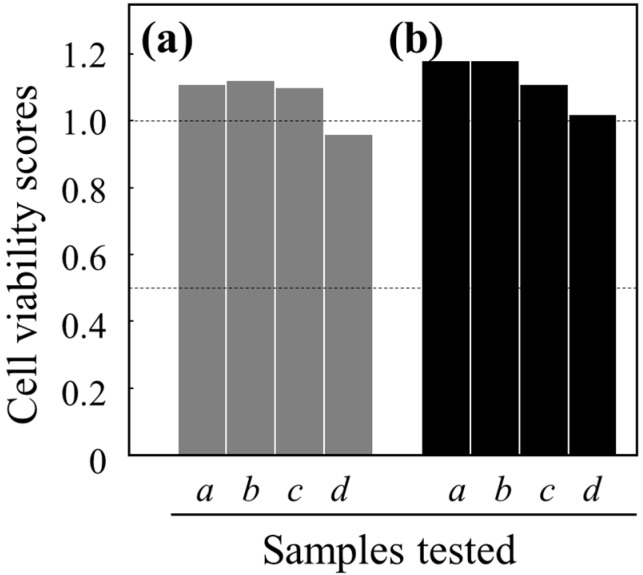
Effect of a PGAIC-based anti-influenza coating agent on the viability (proliferation ability) of Madin–Darby canine kidney (MDCK) cells. (**a**) Non-coated and (**b**) PGA/HDP-coated PET films were subjected to the cytotoxicity tests. Bars *a*, washings from these film surfaces; *b*, 10-fold dilution of *a*; *c*, 100-fold dilution of *a*; *d*, 1000-fold dilution of *a*. PGAIC used as surface-coating materials was thus substantially safe (non-toxic) for human cells.

We have also found that PGA/HDP-coated PET films can be dramatically suppressed the infection of *Influenza A* and *B* viruses (Figure 4) via a contact process. In actuality, the log reduction values (LRVs) against the *A* and *B* viruses were estimated to be 5.4 and 3.2, respectively, while those of non-coated PET films were <0.7. PGA/HDP can be hence characterized as promising antiviral coatings. A higher (probably unacceptable) concentration of HDP salts (e.g., cetylpyridinium chloride) has indeed potential as an antiviral agent [9]; however, it remains to improve its feasibility as antimicrobial coatings. Its higher cytotoxicity [9] is also gaining attention and causes the limited application of cetylpyridinium in pharmaceutics and hygienics. It is thus essential that the PGA/HDP-coated surfaces were virtually non-toxic for human cells (Figure 5).

HDP^+^, a potent and broadly-acting microbicidal agent, comprises a hydrophobic chain (aliphatic alkane) and a hydrophilic ring (pyridinium cation); its hydrophobic chain serves primarily to make initial contact with a cell and attach subsequently to membranes, while its hydrophilic ring increases the permeability of the membrane causing the cytoplasmic contents to leak, resulting in cell death [11,12,13,14,15,16]. Although PGA/HDP is a supra-molecular (bio-based) plastic formed via multiple ionic bonds (Figure 1), the rates of dissociation and diffusion of HDP^+^ from the body are strictly controlled (or limited) [3], allowing for its safe use to human cells (Figure 5). In formulating antimicrobial agents, polymeric materials are generally more efficient and selective (thus safer) than the smaller molecules [17] and facilitate prolonged activity owing to the controlled release of drug moieties from the supra-molecular networks, which can reduce the prevalence of drug-resistant infectors. Because PGA/HDP is easily transformed into a nanofiberplastic [3] in addition to a safe dispersant to create bioactive surfaces, it may contribute to hygienic control and infection prophylaxis in various public facilities such as schools, hospitals, hotels, and transportations, resulting in a decreased risk of airborne infection, contagion, and serious pneumonia, which can be lethal. Moreover, these coatings can also aid against an unforeseen epidemic of new viral infectors. We are now studying on the unique contact-killing mode of PGAIC-coated surfaces and its molecular mechanism.

## 3. Experimental Section

### 3.1. Materials

l-PGA was isolated from the culture media of *Natrialba aegyptiaca* according to published procedures [1]. Partner surfactants, *i.e.*, HDP^+^/DDP^+^/BZT^+^ and BZA^+^, were purchased from Wako Pure Chemicals (Osaka, Japan) and Tokyo Chemical Industry (Japan), respectively. Water-insoluble PGAIC was formed via mixing of a 2 wt % solution of l-PGA with a concentrated solution of a surfactant (corresponding to *ca.* 120 mol % of the carboxyl groups contributed by PGA molecules) [3]. Pellets were collected, washed with water at 60 °C, and lyophilized. *n*-Octadecyldimethyl (3-(trimethoxysilyl) propyl) ammonium (QAS) cation was purchased from Wako. All other chemicals were of analytical grade.

### 3.2. ^1^H-Nuclear Magnetic Resonance (NMR) Spectroscopy

Lyophilized PGAIC sample (6 mg) was dissolved in 0.6 mL of tetradeuterated methanol (methanol-*d*_4_) and analyzed using an AVANCE500 spectrometer (BRUKER, Billerica, MA, USA) operating at 500 MHz. The NMR signals were assigned by analyzing the chemical shifts (ppm) and their relative intensities and coupling patterns in addition to comparisons with the signals of starting materials, *i.e.*, l-PGA and the partner surfactants.

### 3.3. Gel Permeation Chromatography (GPC) Conditions for PGAICs

PGAIC (1 mg) was first dissolved in 1 mL of desterilized ethanol (>99.5 wt %). Fifty µL of the solution was applied to a Shodex Asahipak GF-7M HQ column (300 × 7.5 mm; SHOWA DENKO, Tokyo, Japan) and developed at 30 °C with a mobile phase of ethanol delivered at 0.6 mL·min^−1^. PGAIC was detected at 210 nm using an L-4200 UV detector (Hitachi, Tokyo, Japan). In actuality, the GPC profiles of PGA/HDP and PGA/BZA are represented in Figure S1.

### 3.4. Rapid Coating of PGAIC on Material Surfaces

On plastic surfaces. A test solution of PGAIC (0.1 wt %) was prepared using 70 wt % ethanol. Eighty µL of the solution was sprayed three times at a distance of 5 cm from the surface of a disk (10 mm *dia.*) of a mask made of polypropylene (PP) non-woven fabrics (AS ONE, Osaka, Japan) and the resulting disks were air-dried. To assess the coating performance of PGAICs, the disks were soaked three times in 25 mL of distilled water at 28 °C, dried at 100 °C, and used as PGAIC-coated PP disks. Test solutions were sprayed under the same conditions on metal surfaces, *i.e.*,a stainless steel sheet (SUS304, TOYOBO ENGINEERING, Osaka, Japan; 10 × 10 × 1 mm^3^) and on ceramic surfaces, *i.e.*, a bathroom tile sheet (10 × 10 × 1 mm^3^) (EXTEL HOMES, Gifu, Japan). After the water-soaking and heat-drying treatment, they were applied as PGAIC-coated metal and ceramic sheets, respectively.

### 3.5. Bromophenol Blue (BPB) Staining

BPB is an anionic dye and serves for the detection (determination) of various quaternary ammonium compounds [18,19]. The BPB-staining method [20] was thus applied for the visualization of immobilized cationic surfactants on the surfaces of PGAIC-coated materials. In fact, these materials were soaked in 10 mL of 0.04 *w*/*v* % BPB solution (Nakarai, Kyoto, Japan) for 10 min at 28 °C, then washed in 100 mL of running water, and air-dried. Retained blue colors are virtually indicative of the presence of partner surfactants of PGAIC. Further, QAS-coated materials were used as the control.

### 3.6. Antibacterial Assay

Soybean-casein digest (SCD; Wako) was used for the cultivation of *S. aureus*. The cultures (~5 × 10^4^ CFU) were spread on the plates of SCD agar (Wako) and incubated at 35 °C for 48 h. The antibacterial activity of PGAIC-coated materials was determined using the halo assay described in the Japan Industrial Standards (JIS L 1902) [21].

### 3.7. Antifungal Assay

Cultures of *C. albicans* NBRC1594 (~5 × 10^4^ CFU) were spread on the plates of Sabouraud dextrose agar (Wako) and incubated at 25 °C for 48 h. The antifungal activity [22] of the PGAIC-coated materials was also estimated using essentially the same protocols described above [21].

### 3.8. Antiviral Assay

A concentrated solution of PGAIC (20 wt %) was prepared using ethanol (>99.5 wt %) and applied to the surface of thin films (50 × 50 × 0.188 mm^3^) of polyethylene terephthalate (PET; Toyobo, Shiga, Japan) using a Baker-type Applicator (Tester Sangyo, Saitama, Japan) and dried at 100 °C. The resulting films with a coating thickness of 18 µm were used for the inactivation of *Influenza A* (H1N1, A/PR/8/34) and *B* (B/Shanghai/361/2002) viruses. In reference to JIS Z2801 [22], the virus inoculants (0.2 mL each) were first placed in contact with PGAIC-coated and non-coated PET films at 25 °C for 24 h, and collected into 10 mL of phosphate-buffered saline (PBS; pH 7.0). Their median tissue culture infectious doses (TCID_50_) were determined by the method of Reed and Muench [10]. The LRV scores, where the score of >2.0 is defined to be significant in virus inactivation, were estimated from the comparison with the original (initial) TCID_50_ values of the *A* and *B* inoculants. In fact, the data against *Influenza A* and *B* were obtained using log_10_ (2.1 × 10^7^/TCID_50_ of the sample) and log_10_ (1.7 × 10^5^/TCID_50_ of the sample), respectively.

### 3.9. Cytotoxicity Test

A fifth mL of PBS was placed in contact with PGAIC-coated and non-coated PET films at 25 °C for 24 h, and collected into 10 mL of PBS. The collected solution was diluted 10-, 100-, and 1000 times using PBS to utilize as the samples. Dulbecco’s modified Eagle’s medium (50 µL) containing 5 wt % fetal bovine serum and Madin–Darby canine kidney (MDCK) cells was mixed with the equal volume of the sample and cultivated for 4 days in a CO_2_ incubator. PBS was used for the control. After the cultivation, live MDCK cells were stained using crystal violet and counted. When the score of PBS is defined as 1.0, the subjects displaying those of <0.5 are generally considered as toxic compounds for human cells.

### 3.10. Minimal Inhibition Concentration (MIC) Determination

MIC was determined according to the guidelines of the Clinical and Laboratory Standards Institute (formerly called the National Committee for Clinical Laboratory Standards) [7,8].

## 4. Conclusions

In this study, the supra-molecular plastics of PGAIC were effectively synthesized from archaeal, stereo-regular l-PGA using some cationic surfactants, *viz.* PGA/HDP, PGA/DDP, PGA/BZA, and PGA/BZT. These PGAICs possessed versatile coating performance to create bioactive (anti-staphylococcal and anti-Candida) surfaces on different materials with no additional treatments for the stabilization. Interestingly, PGA/HDP-coated surfaces showed significant anti-influenza activities, while they were safe (non-toxic) for human cells. We are therefore hopeful that PGAICs generate considerable interest among researchers seeking to develop advanced polymer coatings for infection prophylaxis.

## Supplementary Materials

Supplementary materials can be found at http://www.mdpi.com/1422-0067/16/10/24588/s1.
