# Supplementary material for: Poly-γ-glutamate-based Materials for Multiple Infection Prophylaxis Possessing Versatile Coating Performance
Source: Int J Mol Sci. 2015 Oct 15;16(10):24588–99. doi: 10.3390/ijms161024588 (PMC4632766; doi:10.3390/ijms161024588)
Supplement: Supplementary file 1 [file ijms-16-24588-s001.pdf]

Supplementary Information

^{1}H-NMR Analysis of New PGAIC Materials

Figure 2A: PGA/DDP (pyridinium ring (chemical shifts a, b, and c, 9.01, 8.60, and 8.12; relative intensities, 2.13, 1.08, and 2.03), pyridinyl CH_2 (d, 4.65; 2.13), αCH-PGA (α, 4.16; 1.00), γCH_2-PGA (γ, 2.26; 1.89), CH_2-DDP and βCH_2-PGA (e + β, 2.01–1.88; total 4.08), alkane-DDP (f, 1.37–1.27; total 18.51), and CH_3-DDP (g, 0.88; 3.17)).

Figure 2B: PGA/BZA (quaternary ammonium benzyl CH_2 (chemical shifts a and b, 7.56–7.50 and 4.52; relative intensities, 5.17 and 2.00), αCH-PGA (α, 4.18; 1.00), quaternary ammonium CH_2 (d, 7.56–7.50; 2.00), quaternary ammonium CH_3 (c, 3.02; 6.19), γCH_2-PGA (γ, 2.27; 1.80), CH_2-BZA and βCH_2-PGA (e + β, 2.05–1.87; total 3.74), alkane-BZA (f, 1.39–1.28; total 22.64), and CH_3-BZA (g, 0.89; 3.12)).

Figure 2C: PGA/BZT (quaternary ammonium benzyl CH_2 and benzene-BZT (shifts a + a' and b, 7.56–6.81 and 4.61; intensities, total 11.34 and 2.48), αCH-PGA and quaternary ammonium $(CH_2)_2$ (α and d + e, 4.21–3.58; total 11.21), γCH_2-PGA (γ, 2.27; 2.00), βCH_2-PGA (β, 2.05–1.87; 2.23), CH_2-BZT (g, 1.71; 2.61), CH_3-BZT (f, 1.31; 7.63), and tBu-BZT (h, 0.68; 11.06)).

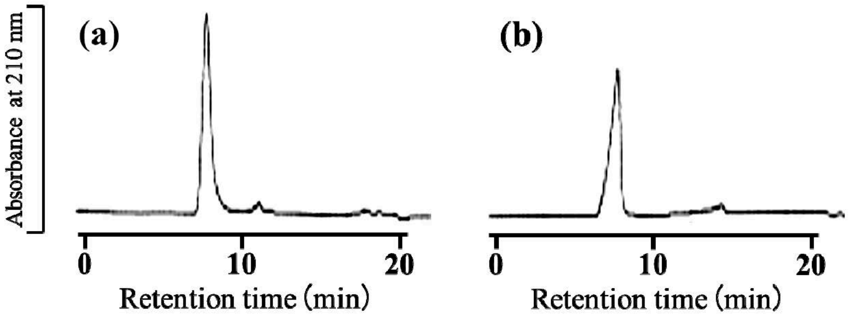

Figure S1. Chromatograms in GPC of (**a**) PGA/HDP and (**b**) PGA/BZA dissolved in ethanol. Estimated retention volumes (mL): PGA/HDP, 4.9; PGA/BZA, 4.8. The results indicated that both the PGAICs are hydrophobic polymers, the molecular weights of which are on average over 1,000,000 [23,24].

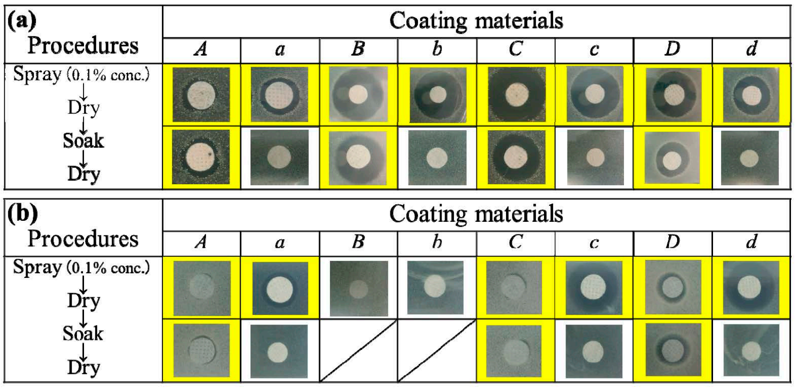

Figure S2. (**a**) Anti-staphylococcal (*bacterial*) and (**b**) anti-Candida (*fungal*) activities of PGAIC-coated PP (*plastic*) disks. Coating materials: images *A*, PGA/HDP; *a*, HDP^+; *B*, PGA/DDP; *b*, DDP^+; *C*, PGA/BZA; *c*, BZA^+; *D*, PGA/BZT; *d*, BZT^+. Antimicrobial performance: yellow panels denote results with halos present around the coated disks; white panels denote results with no halos present around the coated disks.

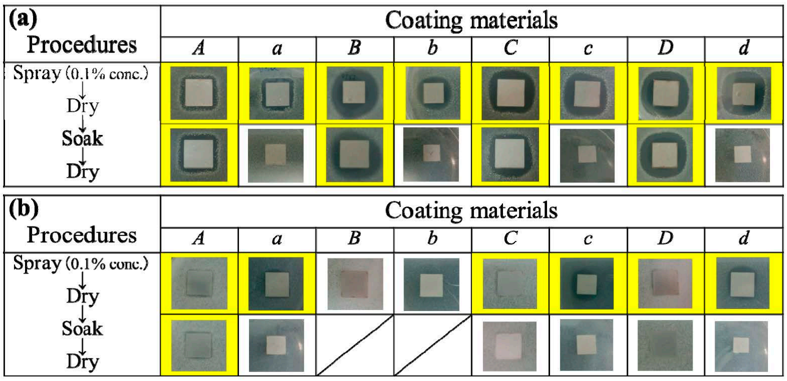

Figure S3. (**a**) Anti-staphylococcal and (**b**) anti-Candida activities of stainless steel (*metal*) sheets. The coating materials were the same as those described in Figure S2. Antimicrobial performance: yellow panels denote results with halos present around the coated sheets; white panels denote results with no halos present around the coated sheets.

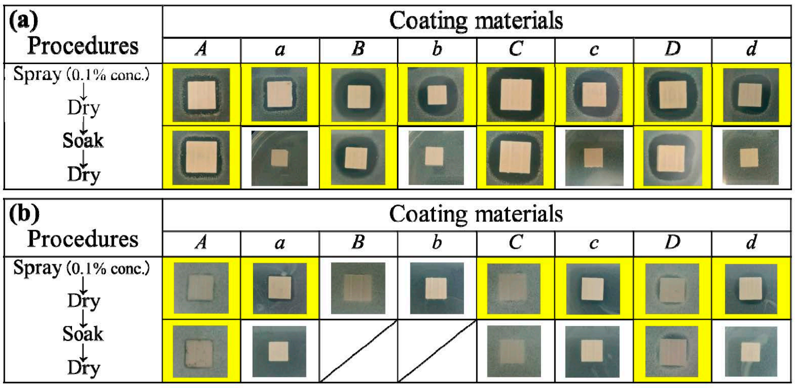

Figure S4. (**a**) Anti-staphylococcal and (**b**) anti-Candida activities of bathroom tile (*ceramic*) sheets. The coating materials were the same as those described in Figure S2. Antimicrobial performance: yellow panels denote results with halos present around the coated tiles; white panels denote results with no halos present around the coated tiles.

(a)

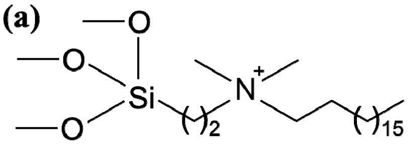

(b) Procedures	Coated materials X	Y	Z
Spray (0.1% conc.) ↓ Dry ↓ Soak ↓ Dry	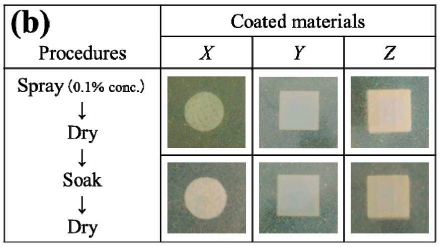		

Figure S5. (**a**) Chemical structure of an *n*-octadecyldimethyl [3-(trimethoxysilyl) propyl] ammonium (QAS) cation and (**b**) anti-staphylococcal activities of QAS-coated materials. Coated materials: images *X*, a PP (*plastic*) disk; *Y*, a stainless steel (*metal*) sheet; *Z*, a bathroom tile (*ceramic*) sheet. The zone of inhibition was not observed around the QAS-coated materials regardless of water-soaking treatment, resulting from its lower anti-staphylococcal activity (Table 2).

© 2015 by the authors; licensee MDPI, Basel, Switzerland. This article is an open access article distributed under the terms and conditions of the Creative Commons Attribution license (http://creativecommons.org/licenses/by/4.0/).
